# Expression of the quinoa *TCP* gene family and analysis of *CqTCP11* involvement in seed germination stress response

**DOI:** 10.3389/fpls.2026.1860606

**Published:** 2026-06-02

**Authors:** Shanmin Zhou, Panpan Shi

**Affiliations:** 1 School of Life Science, Shanxi Normal University, Taiyuan, China; 2 College of Resources and Environmental Sciences, National Academy of Agriculture Green Development, China Agricultural University, Beijing, China

**Keywords:** abiotic stress, CqTCP11, expression pattern, germination, quinoa TCP family

## Abstract

The TCP transcription factor family is a key regulator of plant growth, development and stress adaptation. The TCP gene family in the stress-tolerant cereal *Chenopodium quinoa* has not been systematically characterized. In this study, 20 non-redundant *CqTCP* members were identified genome-wide in quinoa and classified into three subfamilies (PCF, CIN and CYC/TB1) based on phylogenetic relationships. *CqTCPs* possess few introns, and their promoter regions are enriched in various cis-acting elements related to abiotic stress, hormone and light responses. They exhibit tissue-specific expression, and the expression of multiple members is significantly regulated by salt and drought stresses. Functional verification showed that *CqTCP11* is localized in the nucleus. Heterologous overexpression of this gene in *Arabidopsis thaliana* significantly improved seed germination rate under salt and drought stresses, and enhanced the antioxidant capacity and osmotic adjustment ability of seedlings. This study systematically characterized the features of the quinoa TCP family and its functions in stress responses, clarified the key role of *CqTCP11* in stress resistance during seed germination, and provided candidate genes and theoretical support for the genetic improvement of stress resistance in quinoa.

## 1 Introduction

Plant growth and development do not occur in a static environment but require continuous perception and dynamic responses to complex and variable endogenous and exogenous signals. Within this sophisticated regulatory network, transcription factors act as pivotal molecular switches. By specifically binding to cis-acting elements in the promoter regions of target genes, they activate or repress downstream gene transcription, thereby establishing intricate cascading regulatory pathways that ultimately determine cell fate, organ morphology, and overall plant adaptability (Latchman, 1997). Among numerous plant-specific transcription factor families, the TCP (TEOSINTE BRANCHED1/CYCLOIDEA/PCF) family has garnered widespread attention due to its central role in regulating plant architecture, organ size, and environmental adaptation (Martín-Trillo and Cubas, 2010). The family name originates from three representative members: TEOSINTE BRANCHED1 (TB1) in maize, which controls tillering; CYCLOIDEA (CYC) in snapdragon, which determines floral symmetry; and PROLIFERATING CELL FACTORS 1 and 2 (PCF1/PCF2) in rice, associated with cell proliferation (Doebley et al., 1997; Luo et al., 1996). All TCP proteins contain a conserved domain of approximately 59 amino acids. This domain adopts a non-canonical basic helix-loop-helix (bHLH) fold, enabling protein-DNA (recognizing core sequences like GGNCCCAC) or protein-protein interactions (Kosugi and Ohashi, 1997; Manassero et al., 2013; Zhang et al., 2023). Based on subtle sequence variations within the TCP domain and differences in C-terminal regulatory motifs, this family is phylogenetically divided into two ancient and often functionally antagonistic classes: Class I (represented by PCF) and Class II. Class II can be further subdivided into the CYC/TB1 subfamily, primarily involved in floral development and branch control, and the CIN subfamily, mainly regulating leaf growth and senescence (Navaud et al., 2007; Palatnik et al., 2003).

Functioning as “master regulators” of plant growth and development, the functional network of TCP transcription factors spans the entire plant life cycle (Fang et al., 2024; K. Wang et al., 2025). During the vegetative stage, CIN subfamily members are key regulators of leaf morphogenesis. For instance, the barley BDI1 gene encodes a TCP transcription factor that plays a crucial role in determining inflorescence architecture and spikelet development (Shang et al., 2020). In reproductive development, the CYC/TB1 subfamily governs floral organ differentiation and inflorescence structure establishment. The dorsal-specific expression of the snapdragon *CYC* gene determines the formation of zygomorphic flowers (Hileman, 2014), while OsTB1/FC1 in rice regulates tiller number by suppressing axillary bud outgrowth, directly impacting yield components (Takeda et al., 2003). More importantly, the TCP family serves as a critical hub for integrating endogenous hormonal signals with external environmental cues. Studies show that Class I TCP factors (e.g., AtTCP14/15) interact directly with DELLA proteins, key components of the gibberellin (GA) signaling pathway, positively regulating GA-mediated seed germination, hypocotyl elongation, and flowering time (Davière et al., 2014; Resentini et al., 2015). Conversely, Class II CIN-type proteins (e.g., AtTCP4) engage in complex interactions with the jasmonic acid (JA) signaling pathway, mediating trade-offs between leaf development and defense responses (Schommer et al., 2014). Recent research has further revealed the important role of TCPs in plant responses to abiotic stress. In *chrysanthemum*, for example, the organ gigantism phenotype induced by autopolyploidy is associated with significant upregulation of the *CnTCP11* gene, which functions by positively regulating the expression of genes in the downstream GA biosynthesis pathway (Yu et al., 2022). In apple, the MdTCP46-MdABI5-MdEM6/MdRD29A regulatory module plays a key role in ABA signaling and drought stress response, influencing plant drought tolerance by regulating physiological processes such as proline accumulation (Liu et al., 2022). These findings highlight the indispensable regulatory function of TCP transcription factors in coordinating plant growth, development, and stress adaptation.

Quinoa is an annual dicotyledonous plant of the *Chenopodiaceae* family originating from the Andes Mountains. In recent years, it has become a globally renowned “super grain” and a model stress-tolerant crop due to its superior nutritional value and strong environmental adaptability (Angeli et al., 2020). Its seeds are rich in high-quality protein (containing all essential amino acids), dietary fiber, minerals, vitamins, and various health-beneficial secondary metabolites, and are gluten-free, offering a comprehensive nutritional profile (Abugoch James, 2009; Ballegaard et al., 2021; Vega-Gálvez et al., 2010). However, quinoa’s most remarkable characteristic lies in its extraordinary tolerance to multiple extreme abiotic stresses, such as high salinity, drought, low temperature, and poor soil fertility. This enables it to grow and maintain relatively stable yields on marginal lands (e.g., saline-alkali, arid, and semi-arid regions) where other crops struggle, holding significant strategic importance for global food security and climate change adaptation (López-Cervantes et al., 2021; Ren et al., 2023; Sales et al., 2021).

Given the central position of TCP transcription factors in plant biology and the immense potential of quinoa as an important stress-tolerant crop and a new model for functional genomics research, a comprehensive identification and in-depth analysis of the quinoa *TCP* gene family has become an urgent and important task. This study aims to utilize the published quinoa genome data to conduct a genome-wide analysis of the TCP transcription factor family in quinoa. We systematically identified 20 non-redundant CqTCP members and classified them into three subfamilies—PCF, CIN, and CYC/TB1—based on phylogenetic relationships. Gene structure and conserved motif analyses revealed an “intron-poor” characteristic of this family, with structural distribution highly consistent with phylogenetic relationships. Promoter cis-acting element analysis indicated that *CqTCP* genes may be widely involved in abiotic stress responses, hormone signaling, and light reaction regulation. Expression pattern analysis further revealed the tissue-specific expression of *CqTCPs* and their differential regulation under salt and drought stress. Most importantly, this study focused on the *CqTCP11* gene. Subcellular localization confirmed its nuclear localization, and the use of an *Arabidopsis* overexpression system revealed its biological function in significantly promoting seed germination and enhancing seedling stress tolerance under salt and drought conditions. We preliminarily elucidated the mechanism by which *CqTCP11* activates stress-related gene networks to coordinately regulate osmotic balance, reactive oxygen species scavenging, and signal transduction pathways. These results provide an important basis for a deeper understanding of the regulatory functions of TCP transcription factors in quinoa stress adaptation and lay a foundation of candidate gene resources for the genetic improvement of stress tolerance in quinoa.

## 2 Materials and methods

### 2.1 Plant materials and growth conditions

This study used the widely cultivated quinoa variety “YT077” with typical stress-tolerant characteristics as experimental material. Seeds underwent a strict surface sterilization procedure: immersion in 75% (v/v) ethanol for 1 minute, followed by immersion in a sodium hypochlorite solution containing 2% (v/v) available chlorine for 15 minutes, and finally rinsing five times with sterile distilled water. Sterilized seeds were sown in seedling pots containing sterilized substrate. The substrate composition was vermiculite: nutrient soil = 1:3 (volume ratio). The nutrient soil was a mixture of peat, perlite, and vermiculite, with pH adjusted to 6.0 ± 0.2. All plants were uniformly cultured in a growth chamber under strictly controlled conditions: day/night temperatures of 22°C/18°C, a photoperiod of 16 hours light/8 hours dark, light intensity set at 250 μmol·m^−^²·s^−^¹, and relative humidity maintained at 70% ± 5%. From sowing, plants were watered every two days with 1/2 MS nutrient solution, keeping the substrate moist but avoiding waterlogging.

### 2.2 Experimental design for abiotic stress treatments

According to previous studies (Sosa-Zuniga et al., 2017), When seedlings reached 20 days old (four-leaf, one-heart stage), plants with uniform growth status, similar plant height, and leaf number were selected for stress treatment. The experiment used a completely randomized block design, with three biological replicates per treatment, each containing 10 seedlings. Drought stress treatment: mannitol was used to simulate drought stress. Accurately weigh mannitol powder and dissolve it in 1/2 MS nutrient solution to prepare a 100 mM mannitol treatment solution. Treatment was applied via root irrigation, ensuring complete substrate penetration. The control group was irrigated with an equal amount of 1/2 MS solution. Salt stress treatment: Sodium chloride was used to simulate salt stress. A 100 mM NaCl stress solution was prepared using 1/2 MS nutrient solution and similarly applied via irrigation. Sampling time points: Samples were collected at two time points: 0 and 6 hours after the start of treatment. At each time point, five seedlings were randomly selected from each biological replicate. Using liquid nitrogen pre-cooled forceps and scissors, roots and the second pair of true leaves from the shoot were quickly separated. Samples were immediately frozen in liquid nitrogen and then transferred to a -80°C ultra-low temperature freezer for long-term storage until RNA extraction.

### 2.3 Identification of the *CqTCP* gene family

The latest quinoa reference genome sequence file and gene structure annotation file were downloaded from the Phytozome database (https://phytozome-next.jgi.doe.gov/) (Goodstein et al., 2012). For *Arabidopsis thaliana*: The full-length sequences of all 24 annotated AtTCP proteins were downloaded from the TAIR database (https://www.arabidopsis.org/) (Garcia-Hernandez et al., 2002). A multi-step candidate gene identification strategy employing “homology alignment + domain scanning” was adopted to ensure comprehensiveness and accuracy. First, local BLASTP search: Using the NCBI BLAST + 2.13.0 software package, all *Arabidopsis* TCP protein sequences were used as query sequences to perform a local BLASTP search against the quinoa genome-encoded protein database, retaining all hit sequences. Then, HMMER domain scanning: The characteristic hidden Markov model file “PF03634” for the TCP family was downloaded from the Pfam database (https://pfam.xfam.org/) (Mistry et al., 2021; Paysan-Lafosse et al., 2025). Using HMMER 3.3.2 software, the hmmsearch command was used to scan the quinoa whole proteome, retaining all protein sequences containing the complete TCP domain. Finally, candidate sequence integration and verification: Sequences obtained from the two methods were combined to generate a preliminary candidate list. The online batch search function of the NCBI Conserved Domain Database (CDD, https://www.ncbi.nlm.nih.gov/cdd) and the SMART database (http://smart.embl-heidelberg.de/) were used to re-verify the domain architecture of candidate protein sequences (Lei et al., 2022). Only protein sequences confirmed by both databases as containing the typical TCP domain (PF03634) were retained as final members of the *CqTCP* gene family. The nomenclature of the *CqTCP* gene family follows the previous naming convention (Bakhtari et al., 2024).

### 2.4 Sequence characterization and physicochemical property analysis

Protein physicochemical properties: All finalized CqTCP protein sequences were submitted to the ExPASy ProtParam tool (https://web.expasy.org/protparam/) to batch calculate parameters for each protein: number of amino acids, molecular weight (Da), theoretical isoelectric point (pI), grand average of hydropathicity (GRAVY) value, instability index, etc. Subcellular localization prediction: Three online tools were used for prediction, and the majority consensus result was taken as the final predicted localization: WoLF PSORT II (https://wolfpsort.hgc.jp/); Plant-mPLoc (http://www.csbio.sjtu.edu.cn/bioinf/plant-multi/); Cell-PLoc 2.0 (http://www.csbio.sjtu.edu.cn/bioinf/Cell-PLoc-2/). Ka/Ks molecular evolutionary selection analysis was performed following the previous method (Bakhtari & Zamani, 2025).

### 2.5 Phylogenetic tree construction and classification

Full-length protein sequences from quinoa and *Arabidopsis* were combined. Multiple sequence alignment was performed using the ClustalW2 program built into MEGA7.0 software. The alignment result was imported into MEGA7.0 for phylogenetic tree construction using the Neighbor-Joining (NJ) method. Gaps were handled by partial deletion, with a site coverage cutoff of 95%. The Bootstrap method was used to test branch reliability, with 1000 replication cycles. Only branches with Bootstrap support >50% are shown. Classification and Naming: Based on the tree topology and referencing the classic classification criteria for TCP families in *Arabidopsis*, CqTCPs were divided into Class I and Class II, with Class II further subdivided into CYC/TB1 and CIN subfamilies. Systematic naming was performed according to their phylogenetic relationship proximity with *Arabidopsis* homologous genes in the tree. Visualization: The generated tree file was uploaded to the iTOL online platform (https://itol.embl.de/) for beautification and annotation (Letunic and Bork, 2024).

### 2.6 Gene structure and protein conserved motif analysis

Exon-intron structure: Using the GFF3 annotation file downloaded from Phytozome, the genomic DNA sequence and its corresponding CDS (coding sequence) for each *CqTCP* gene were extracted. The “Gene Structure View” function in TBtools software was used to automatically identify exon and intron positions by aligning CDS with genomic sequences (C. Chen et al., 2023) and generate structural schematics. Conserved motif (Motif) identification: The full-length sequences of all CqTCP proteins were submitted to the MEME Suite website (https://meme-suite.org/meme/tools/meme) (Bailey et al., 2015). Parameters were set as follows: maximum number of motifs: 15; motif width range: 6–100 amino acid residues; number of motifs that can occur per sequence: zero or one per sequence; other parameters were kept at default. After completion, the distribution patterns of identified conserved motifs were visualized according to the branch order of the phylogenetic tree. The conserved domains were obtained from the NCBI Conserved Domain Database (CDD).

### 2.7 Expression pattern analysis

Analysis of tissue expression profiles based on public transcriptome data: quinoa-related publicly available transcriptome data were downloaded from the NCBI Sequence Read Archive (SRA) database. Key projects included: PRJNA394651, covering different tissues such as root, stem, leaf, flower, and seed. Data processing pipeline: Fastp v0.23.2 was used for quality control and filtering of raw sequencing reads; HISAT2 v2.2.1 was used to map clean reads to the quinoa reference genome; StringTie v2.2.1 was used for transcript assembly and expression quantification; TPM values from the output were used for subsequent analysis. TPM values for all *CqTCP* genes across samples were extracted, log2-transformed using the “Heatmap” function in TBtools, and hierarchical clustering was performed using Euclidean distance and complete linkage method to draw tissue-specific expression heatmaps.

### 2.8 Validation by real-time quantitative PCR

Total RNA extraction and gene expression measurement were performed according to a previous method (Yu et al., 2024). Briefly, total RNA was extracted from roots and leaves, followed by detection of RNA concentration, purity, and integrity. Then, first-strand cDNA synthesis was performed, and the product was diluted 5-fold and stored at -20°C. Specific primers were designed in low-conservation regions of the *CqTCP* genes using Primer Premier 6.0 software; primer sequences are listed in **Supplementary Table 1**. qRT-PCR reactions and data acquisition were performed using a QIAGEN Rotor-Gene Q real-time PCR machine, with three technical replicates per sample. Ct values were automatically calculated using the instrument’s software (Rotor-Gene Q Series Software 2.3.4). The 2^−ΔΔCt^ method was used to calculate the relative expression of target genes relative to the reference gene, normalized to the expression level of the 0-hour control sample set as 1. Data visualization and statistical analysis were performed using GraphPad Prism 9 software (Mitteer and Greer, 2022).

### 2.9 Subcellular localization

Vector construction: The complete coding sequence (without stop codon) of the selected key gene *CqTCP11* was cloned via homologous recombination into the *pCAMBIA1300-35S-GFP* vector to construct the *35S::CqTCP11-GFP* fusion expression vector. The recombinant vector was introduced into *Agrobacterium tumefaciens* GV3101, which was subsequently used for transient transformation of tobacco epidermal cells. After 48 hours of incubation, observation and imaging were performed using a laser scanning confocal microscope.

### 2.10 Determination of physiological indexes of quinoa

Approximately 0.3 g fresh weight of leaf tissue was weighed and ground in liquid nitrogen with 5% trichloroacetic acid (TCA) for extraction. The centrifuge tube was placed at 4 °C and centrifuged at 10,000 rpm for 15–20 minutes to obtain the supernatant. A 2 mL aliquot of the extract was mixed with 2 mL of TCA solution containing 0.67% thiobarbituric acid (TBA), and reacted precisely in a boiling water bath for 15 minutes, followed by rapid cooling. After the reaction, the tube was immediately placed in an ice-water bath to cool quickly to room temperature, then centrifuged at 4000 rpm for 10 minutes to remove any possible precipitate. Using a sample without TBA as a blank control, the absorbance of the reaction solution at 532 nm and 600 nm was measured with a spectrophotometer to calculate malondialdehyde (MDA) and soluble sugar content.

Free proline in plant tissues was extracted using sulfosalicylic acid. Steps: 0.4 g of tissue was chopped into a test tube, 5 mL of 3% sulfosalicylic acid was added, the tube was capped, and extraction was performed in a boiling water bath for 10 minutes. After rapid cooling to room temperature, the mixture was filtered. A 2 mL aliquot of the supernatant was transferred to another test tube, to which 2 mL of glacial acetic acid and 3 mL of 2.5% acidic ninhydrin reagent were added. The tube was capped, heated in a boiling water bath for 40 minutes, then rapidly cooled. After cooling, 5 mL of toluene was added, mixed thoroughly, and allowed to stand for layer separation. The upper layer was collected to measure absorbance at 520 nm, and proline content was calculated.

Peroxidase (POD) activity was determined using the guaiacol method. 0.4 g of tissue was chopped, and 5 mL of 20 mmol·L^−^¹ KH_2_PO_4_ was added for grinding into a homogenate. The homogenate was centrifuged at 3000 rpm for 5 minutes, and the supernatant was retained. 4 mL of 20 mmol·L^−^¹ KH_2_PO_4_ was added to resuspend the pellet, centrifuged again, and the supernatants were combined and diluted to a final volume of 4 mL as the enzyme extract. For the zero-control group, 0.5 mL of 20 mmol·L^−^¹ KH_2_PO_4_ and 3 mL of reaction mixture were added; for the test group, 0.5 mL of enzyme extract and 3 mL of reaction mixture were rapidly mixed. Immediately, a stopwatch was started, and absorbance at 470 nm was measured every 30 seconds, reading for 5 minutes to calculate POD activity.

### 2.11 Germination rate assay

The full-length coding sequence (CDS) of the *CqTCP11* gene, excluding the stop codon, was cloned into the *pCAMBIA1300-35S-GFP* vector via homologous recombination to construct the *35S::CqTCP11-GFP* fusion expression vector. The recombinant vector was introduced into *Agrobacterium tumefaciens* strain GV3101, which was then used for floral dip transformation of *Arabidopsis thaliana tcp10-1*(SALK_050423.50.65) mutant inflorescences. After seed harvesting and screening, two independent homozygous T3 transgenic lines (*35S-CqTCP11#1* and *35S-CqTCP11#2*) were obtained. These lines, together with *tcp10–1* and *tcp10-2* (SALK_050436.54.25), were used for subsequent experiments. *Arabidopsis* seed germination rate was determined according to a previously described method (X. Chen et al., 2023). Before sowing, seeds were subjected to surface sterilization and vernalization, followed by normal cultivation. Seedlings were grown under strictly controlled conditions: a 16 h light (light intensity 120 μmol m^-2^ s^-1^, 23°C)/8 h dark (22°C) photoperiod and 60% relative humidity. Starting from the second day of cultivation, observations were made under a stereomicroscope at a fixed time each day. Radicle protrusion through the seed coat was used as the criterion for germination, and the number of germinated seeds in each replicate group was accurately recorded. The final germination rate was calculated after 7 consecutive days of recording.

## 3 Results

### 3.1 Genome-wide identification and basic characterization of the *TCP* gene family in quinoa

Through systematic retrieval of the quinoa (Chenopodium quinoa) genome sequence and conserved domain verification, this study identified a total of 20 non-redundant *TCP* transcription factor genes, named *CqTCP1* to *CqTCP20* according to their chromosomal positions (**Supplementary Figure 1**; **Supplementary Table 1**). Analysis of the physicochemical properties of the proteins encoded by these genes showed that their amino acid numbers ranged from 187 to 521, with molecular weights between 20.8 kDa and 56.7 kDa. The predicted isoelectric points (pI) ranged from 5.79 to 10.73, with an average of 8.24, indicating that most CqTCP proteins are basic proteins (**Table 1**). Subcellular localization prediction showed that except for *CqTCP1* predicted to localize to the chloroplast and *CqTCP3* predicted to localize to the cytoplasm, the remaining 18 members were primarily predicted to localize to the nucleus, consistent with their function as transcription factors.

**Table 1:** Basic information of quinoa *TCP* gene family members.

Sequence ID	Number of amino acid	Molecular weight	Theoretical pI	Instability index	Aliphatic index	Grand average of hydropathicity	Location
CqTCP10	556	59692.64	5.97	51.94	63.22	-0.659	nucleus
CqTCP12	318	32483.23	9.9	56.98	63.08	-0.334	nucleus
CqTCP13	283	30551.27	7.92	41.73	56.86	-0.76	nucleus
CqTCP14	184	19933.46	10.12	63.63	66.36	-0.432	nucleus
CqTCP15	246	26333.99	8.97	47.6	59.84	-0.614	nucleus
CqTCP16	221	24248.02	6.71	60.78	63.21	-0.639	nucleus
CqTCP17	514	55593.79	8.54	37.37	74.65	-0.39	nucleus
CqTCP18	293	31325.87	7.91	59.48	62.97	-0.68	nucleus
CqTCP19	297	32534.88	6.83	58.36	70.98	-0.92	nucleus
CqTCP20	240	25389.68	6.01	42.55	69.62	-0.506	nucleus
CqTCP7	192	21647	8.52	54.77	65	-0.764	chloroplast
CqTCP6	359	40788.22	6.84	57.75	70	-0.855	nucleus
CqTCP3	274	30841.24	6.05	57.88	75.36	-0.694	cytoplasm
CqTCP4	208	23189.22	9.02	42.95	68.08	-0.673	nucleus
CqTCP5	404	43310.14	8.81	46.36	54.83	-0.931	nucleus
CqTCP2	365	38903.58	6.43	52.37	61.32	-0.677	nucleus
CqTCP8	393	42577.03	7.47	49.48	64.12	-0.66	nucleus
CqTCP1	442	46588.66	7.76	44.73	58.53	-0.701	nucleus
CqTCP11	331	35857.58	8.87	40.09	57.76	-0.649	nucleus
CqTCP9	293	31521.58	7.1	39.82	52.59	-0.725	nucleus

### 3.2 Phylogenetic analysis and subfamily classification

**Figure 1:**
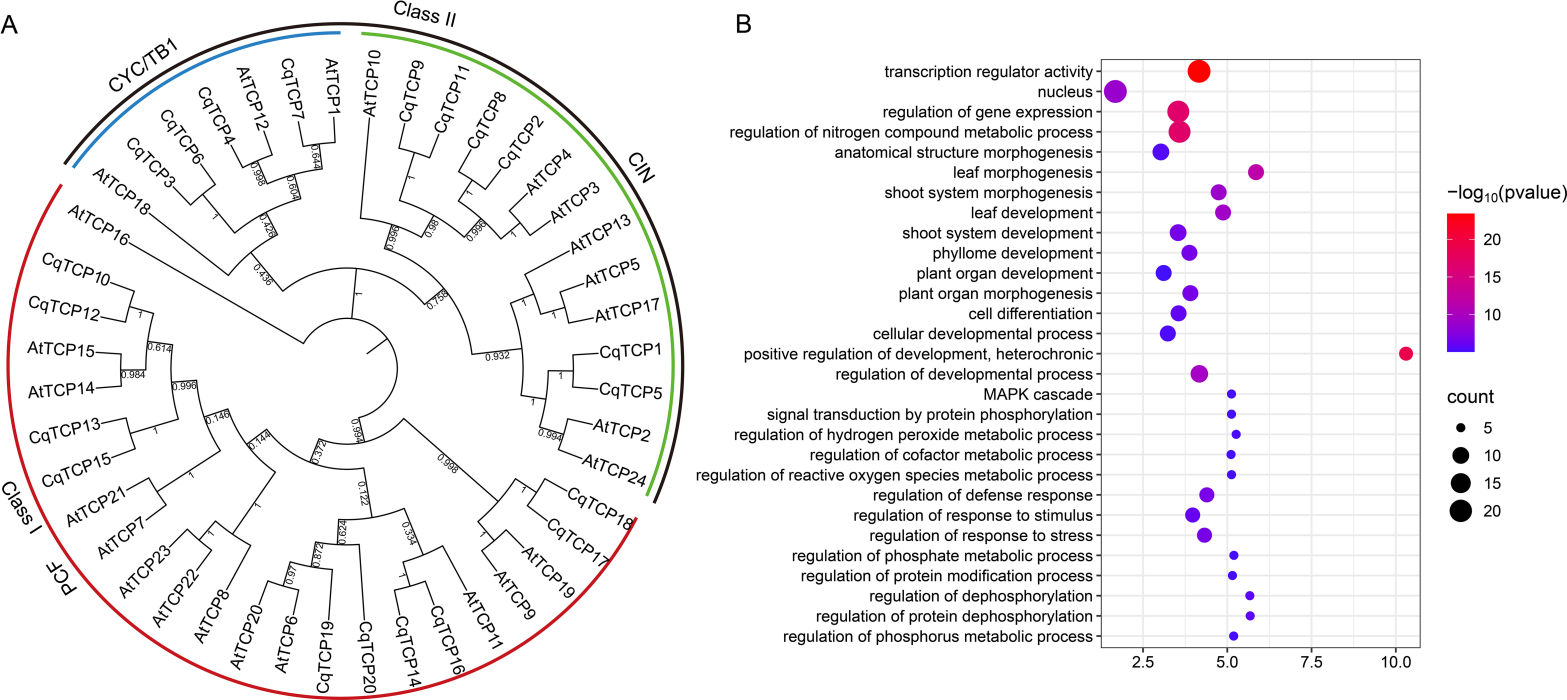
Phylogeny and function of the quinoa TCP family. Phylogenetic tree constructed based on full-length protein sequences of TCP transcription factor family members in quinoa **(A)**. The tree was built using the Neighbor-Joining (NJ) method, with the Poisson model employed for sequence alignment. Bootstrap analysis with 1000 replicates was conducted to assess branch support. TCP protein sequences of *Arabidopsis thaliana* were adopted as reference sequences for subfamily classification. *CqTCP* members are clustered into Class I (PCF subfamily) and Class II (further divided into CIN and CYC/TB1 subfamilies) based on their sequence similarity. Different colored areas delineate the grouping ranges of the various TCP subfamilies. Node values indicate Bootstrap support rates (only values ≥50% are shown). Gene ontology (GO) enrichment analysis of differentially expressed genes **(B)**. The bubble chart displays significantly enriched biological processes (*p* < 0.05), with the x-axis representing the enrichment score (-log_10_*p-value*) and point size corresponding to the number of genes enriched in each term. Terms are broadly categorized into development/morphogenesis.

To elucidate the phylogenetic relationships of CqTCP proteins, this study constructed a Neighbor-Joining phylogenetic tree containing 20 CqTCP proteins from quinoa and 24 AtTCP proteins from *Arabidopsis* (**Figure 1A**). Based on clustering relationships and the classification standards for *Arabidopsis* TCPs, all CqTCP members were clearly divided into three subfamilies: the PCF subfamily (11 members, 55%), the CIN subfamily (6 members, 30%), and the CYC/TB1 subfamily (3 members, 15%). The PCF subfamily had the largest number of members, suggesting it may play broad roles in quinoa fundamental life activities. Similar to the division of the quinoa *ACS* gene family into four groups, the subdivision of TCP subfamilies provides an evolutionary basis for exploring functional differentiation among members. The GO enrichment analysis reveals that the differentially expressed genes are significantly enriched in biological processes related to plant development regulation and stress response (**Figure 1B**). The enriched terms can be classified into two major functional categories: First, processes associated with development and morphogenesis, such as “leaf morphogenesis,” “shoot system morphogenesis,” “plant organ development,” and “cell differentiation,” indicating that these genes play important roles in the formation and development of leaf and aboveground tissues. Concurrently, the enrichment of terms including “transcriptional regulator activity” and “nucleus” suggests that their regulatory functions are primarily exerted at the transcriptional level.Second, signal transduction and stress response processes, including “MAPK cascade,” “signal transduction by protein phosphorylation,” “regulation of reactive oxygen species metabolic process,” “regulation of defense response,” and “regulation of phosphate metabolic process.” These processes are closely associated with molecular responses to biotic or abiotic stresses, particularly through the regulation of protein phosphorylation and dephosphorylation pathways. In summary, this set of genes not only plays a central role in plant organ development and cell differentiation but is also broadly involved in internal and external signal transduction and stress adaptation, implying their potential importance in the cross-regulatory network linking development and environmental adaptation.

### 3.3 Gene structure and conserved motif analysis

**Figure 2:**
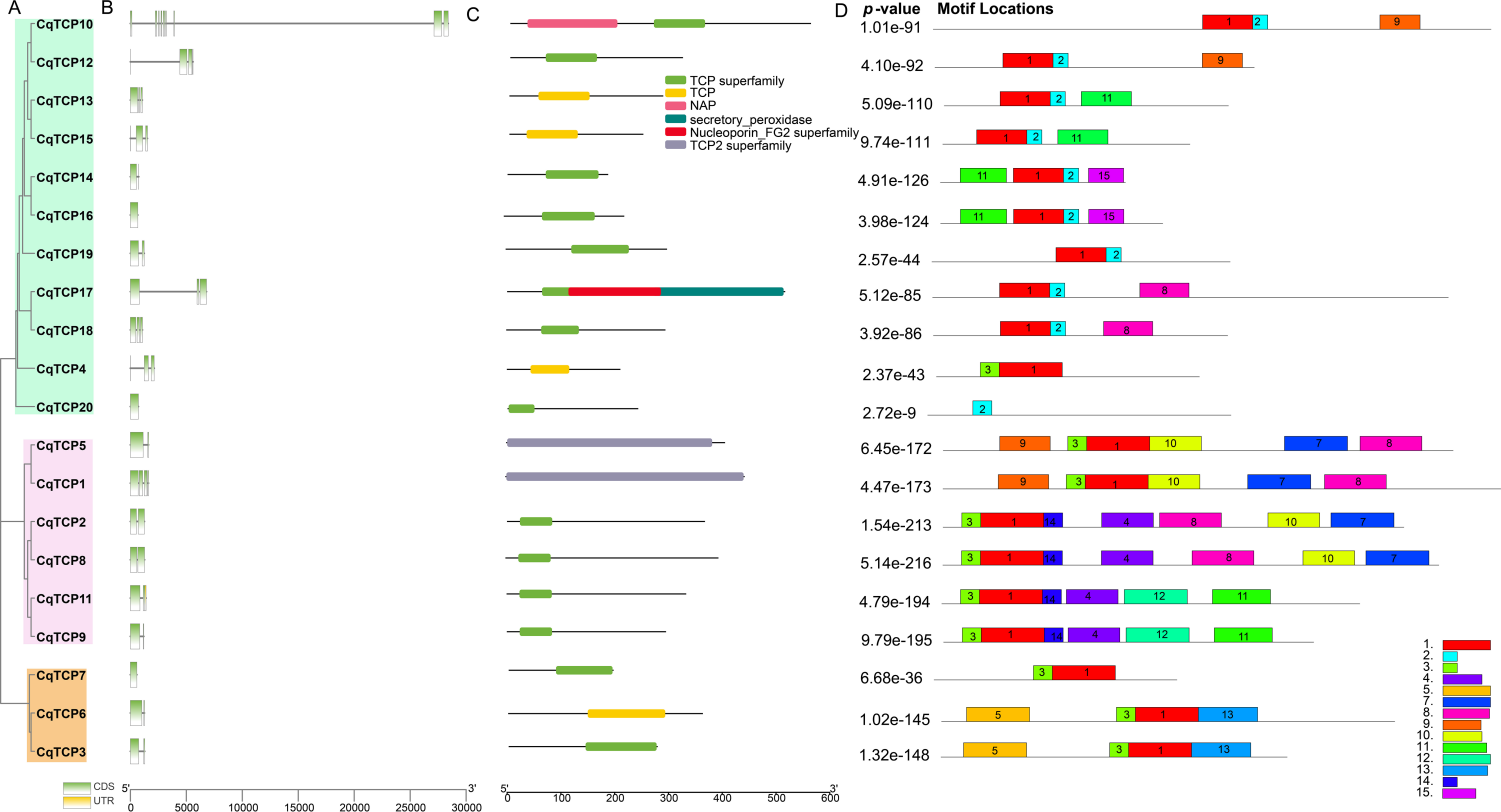
Gene structure of the quinoa TCP family. Gene structure and distribution of conserved protein motifs of TCP transcription factor family in quinoa. **(A)** The left-side phylogenetic tree is based on full-length TCP protein sequences, showing CqTCP members divided into Class I (PCF subfamily) and Class II (CIN and CYC/TB1 subfamilies). **(B)** The middle part shows gene structure schematics, where green boxes represent exons, yellow lines represent introns, and gray lines represent untranslated regions (UTRs). **(C)** Conserved domain distribution of quinoa TCP proteins; different colors denote distinct functional domains. **(D)** The right side shows the distribution map of conserved motifs, with different colored boxes representing the 15 conserved motifs (Motif 1-15) identified using the MEME software.

Gene structure analysis revealed that the exon-intron structures of *CqTCP* family members were relatively simple (**Figures 2A, B**). Ninety percent of the genes have relatively short introns, while *CqTCP7*, *CqTCP16* and *CqTCP20* contain no introns. This “intron-poor” feature is similar to many stress-responsive genes and may facilitate rapid transcription and induced expression in response to environmental stress. Gene structures showed variations in exon number (ranging from 3 to 6) and intron length among different *CqTCP* members, with members within the same evolutionary branch typically sharing similar structural patterns. The Ka/Ks ratios of gene pairs in the CqTCP family are overall much less than 1, indicating that these genes are mainly constrained by purifying selection, and most gene pairs show characteristics of high sequence (**Supplementary Table 1**). Conserved domain and motif analysis revealed that most conserved motifs are located within conserved domains (**Figures 2C, D**). Except for CqTCP20, all other CqTCP member proteins contained Motif 1, which constitutes the core of the TCP domain. Different subfamilies exhibited significant characteristics in motif composition: PCF subfamily members typically contained Motif 1, 2; CIN subfamily members generally contained Motif 1, 6. The distribution differences of specific motifs (e.g., Motif 3) might be related to their functional specificity. Some members contained subfamily-specific motifs (e.g., Motif 5 was present only in *CqTCP2* and *CqTCP3*). The distribution patterns of gene structure and conserved motifs were highly consistent with the evolutionary relationships, collectively revealing the structural differentiation and functional evolution of the TCP family in quinoa.

### 3.4 Promoter cis-acting element analysis

**Figure 3:**
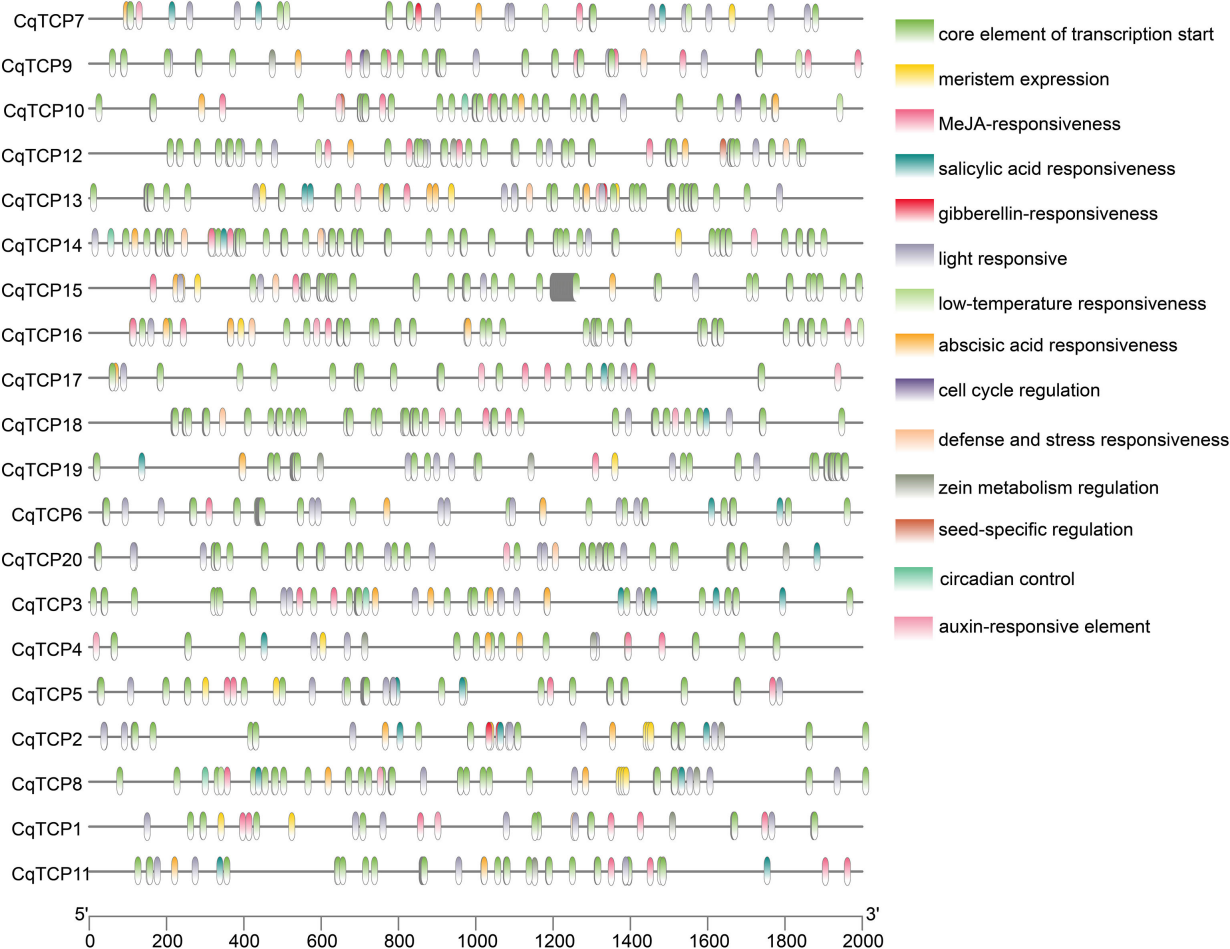
Promoter analysis of quinoa *TCP* family genes. Distribution of cis-acting elements in promoter regions of TCP transcription factor family members in quinoa. For the 2000 bp sequence upstream of the transcription start site (ATG) of each *CqTCP* gene, prediction and identification of cis-acting elements were performed using the PlantCARE database. In the image, blocks of different colors and shapes represent different types of regulatory elements, categorized according to their main functions.

Analysis of the 2000 bp promoter region upstream of the start codon of *CqTCP* genes revealed an abundance of cis-acting elements related to abiotic stress response, plant hormone signal transduction, and light response (**Figure 3**). Mainly included: Stress-responsive elements: Abscisic acid-responsive element (ABRE, present in 12 genes), anaerobic-responsive element (ARE, present in 15 genes), low-temperature-responsive element (LTR), and defense and stress-responsive element (TC-rich repeats). Hormone-responsive elements: Auxin-responsive elements (TGA-element, AuxRR-core), gibberellin-responsive elements (GARE-motif, P-box), methyl jasmonate-responsive elements (CGTCA-motif, TGACG-motif), and ethylene-responsive element (ERE). Light-responsive elements: Various light-responsive elements (e.g., G-box, Box 4, GT1-motif) were widely present. These elements indicate that the expression of *CqTCP* genes may be precisely regulated by various environmental signals and internal hormone networks, and they may particularly play important roles in responses to abiotic stresses such as drought, low temperature, and heavy metals.

### 3.5 Expression patterns of *CqTCP* genes in different tissues and under abiotic stress

**Figure 4:**
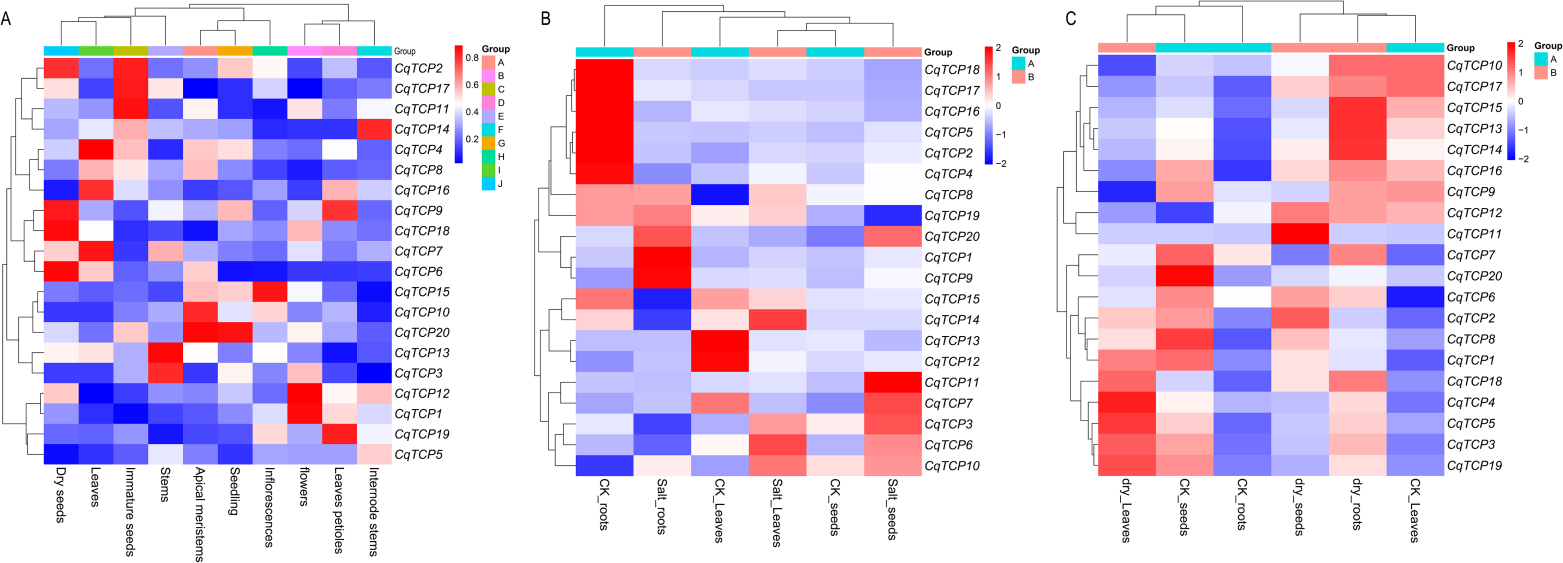
Expression profiles of *CqTCP* genes in quinoa across different tissues and under salt and drought stresses. **(A)** Expression levels of *CqTCP* genes in nine different tissues or developmental stages of quinoa. Heatmap color intensity represents log2-transformed TPM values, with red indicating high expression and blue indicating low expression. **(B, C)** qRT-PCR analysis of relative expression changes of *CqTCP* genes in quinoa seeds, seedling roots, and leaves treated with 1/2 MS medium or 1/2 MS medium supplemented with 100 mM mannitol **(B)** or NaCl **(C)** for 6 hours. Using quinoa *Tubulin* gene as the internal reference, data are presented as mean ± standard deviation.

To explore the potential functions of *CqTCP* genes, public transcriptome data were used to analyze their expression patterns in different tissues such as root, stem, leaf, flower, and seed (**Figure 4A**). Results showed that *CqTCPs* exhibited obvious tissue-specific expression. For example, *CqTCP7* and *CqTCP12* were highly expressed in flowers; *CqTCP11* and *CqTCP17* were specifically highly expressed in mature and immature seeds; while *CqTCP3* showed relatively high expression in leaves and petioles, suggesting its function might be related to leaf development and photosynthesis, respectively. qRT-PCR was further used to analyze the expression of the *CqTCP* gene family under high salt stress (**Figures 4B, C**). It was found that the expression of multiple *CqTCP* genes was significantly induced or repressed by salt stress. For example, *CqTCP4* and *CqTCP18* showed significant upregulation in roots after treatment, and *CqTCP11* expression increased substantially in seeds under salt stress, indicating they may participate in the early salt stress response in quinoa roots. Furthermore, qRT-PCR was used to analyze the relative expression levels of *CqTCP* genes in quinoa seedling roots and leaves at different time points after simulated drought. The study found that *CqTCP4* and *CqTCP18* were upregulated in leaves, and unexpectedly, *CqTCP11* was also upregulated in seeds under drought stress. These differential expression patterns suggest that different *CqTCP* members may play distinct spatiotemporal roles in quinoa physiological processes for coping with salt and drought stress, jointly regulating its drought tolerance. Among them, *CqTCP11* may play an important role in seed response to salt and drought stress, but further research is needed.

### 3.6 *CqTCP11* localizes to the nucleus

**Figure 5:**
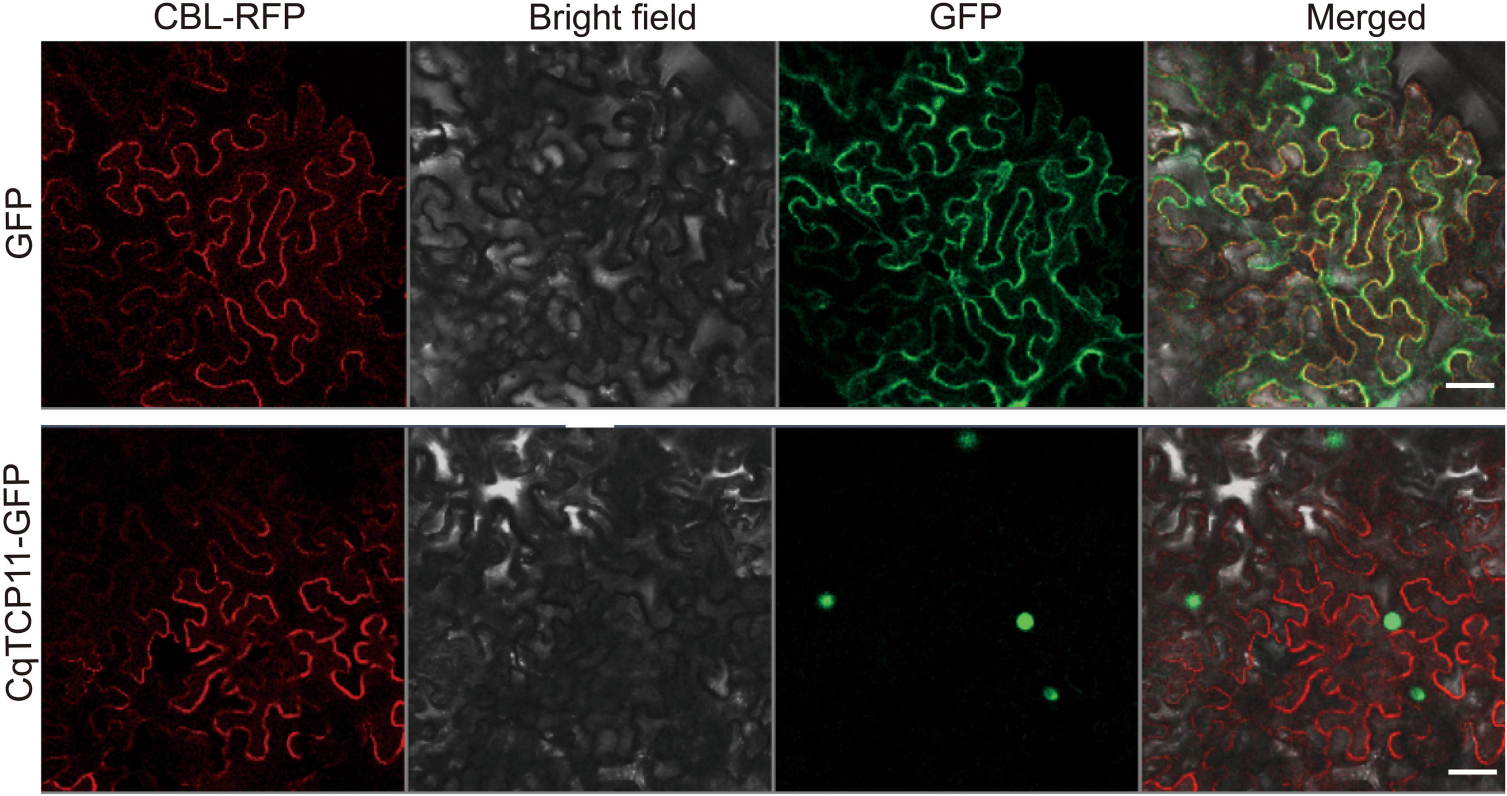
Subcellular localization of *CqTCP11.* Subcellular localization of CqTCP11 protein in tobacco epidermal cells. *35S:GFP* was used as control for *tobacco* epidermal cell transformation; scale bar = 50 µm.

To further investigate the role of *CqTCP11* in seed response to salt and drought stress and to clarify the subcellular distribution of the CqTCP11 protein, this study constructed a *35S:CqTCP11-GFP* fusion expression vector and transiently expressed it in tobacco epidermal cells. Cells transformed with the *35S:GFP* empty vector served as the control. Observation under a laser scanning confocal microscope showed that the green fluorescent signal in the control empty vector group was uniformly distributed throughout the entire cell, including the nucleus, cytoplasm, and cell membrane, indicating that free GFP protein can diffuse freely within the cell. In contrast, the green fluorescent signal of *35S:CqTCP11-GFP* showed a highly concentrated distribution pattern, specifically localized to the nuclear region, with no obvious fluorescent signal detected in the cytoplasm or cell membrane. The subcellular localization results clearly showed that CqTCP11 protein is a nuclear-localized protein (**Figure 5**). This localization characteristic is consistent with its predicted function as a transcription factor, providing direct cytological evidence for its gene transcriptional regulation function within the nucleus.

### 3.7 *CqTCP11* enhances seed germination under salt stress

**Figure 6:**
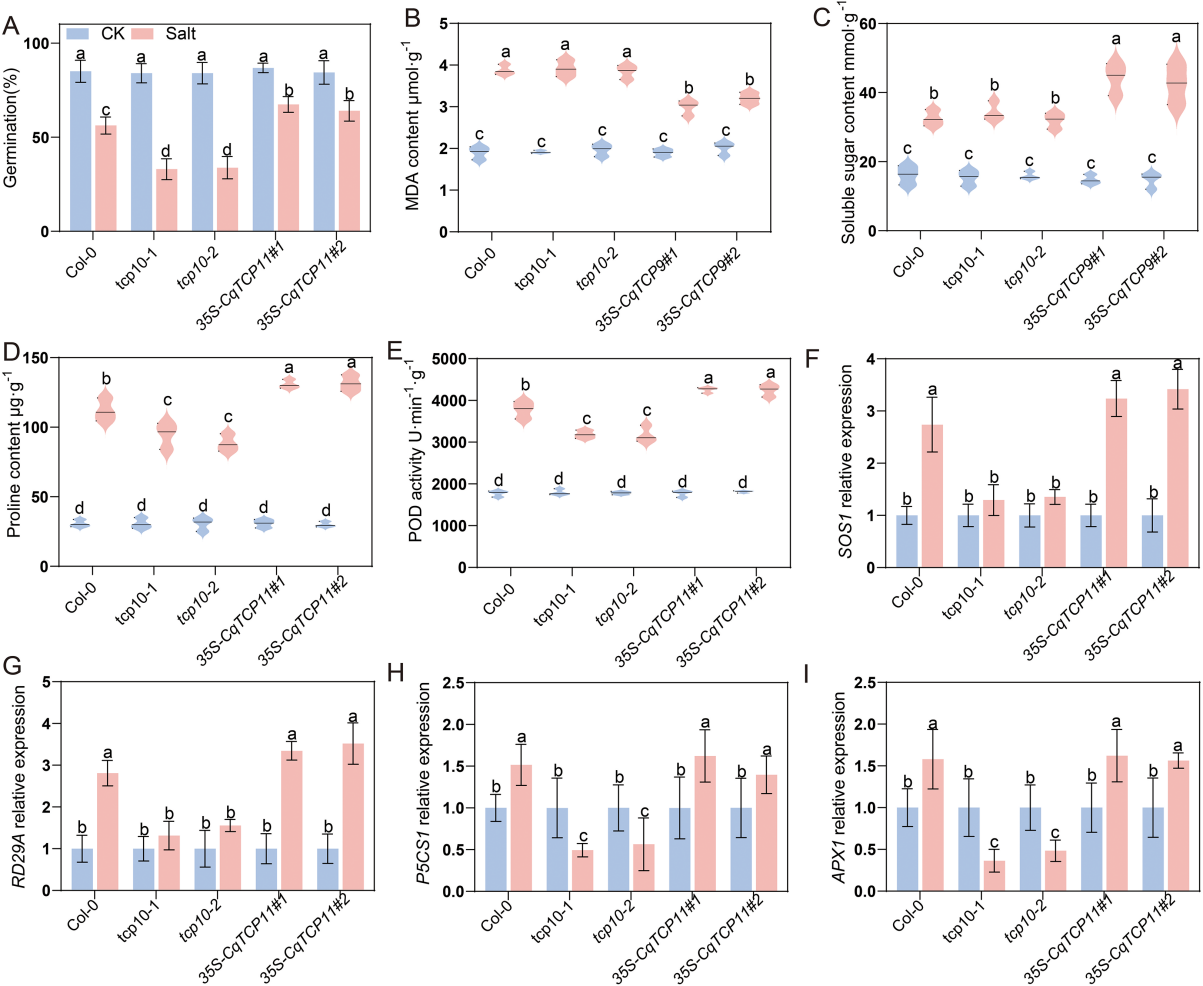
Involvement of *CqTCP11* in quinoa seed salt stress tolerance. **(A)** Germination rates of *Arabidopsis* seeds of wild-type (WT), *tcp10-1*, *tcp10-2*, *35S-CqTCP11#1*, and *35S-CqTCP11#2* lines on 1/2 MS medium with or without 100 mM NaCl were counted after 7 days of culture under 16h light/8h dark (23 °C/20 °C) conditions. Data represent the mean ± SD of at least five independent replicate experiments. **(B-E)** Measurement of malondialdehyde (MDA) content **(B)**, soluble sugar **(C)**, proline (Pro) accumulation **(D)**, and peroxidase (POD) activity **(E)** in seedlings after salt stress (100 mM NaCl treatment for 3 days). **(F-I)** qRT-PCR analysis of the expression of several stress-related genes after 6 hours of salt stress. At least three independent replicate experiments were performed. Different lowercase letters indicate significant differences at *P* < 0.05 based on one-way ANOVA and Tukey’s *post-hoc* test.

To further explore the biological function of *CqTCP11* in abiotic stress responses, we constructed a *CqTCP11* overexpression vector driven by the CaMV 35S strong promoter and obtained homozygous overexpression transgenic lines (*OE-1* and *OE-2*) in *Arabidopsis* via floral dip transformation. Seeds of wild-type (WT), *tcp10-1*, *tcp10-2*, *35S-CqTCP11#1*, and *35S-CqTCP11#2* lines were evenly sown on 1/2 MS solid medium containing different stress agents, cultured under standard light conditions, and their germination rates and radicle elongation were statistically analyzed. Under 100 mM NaCl stress, the germination advantage of *OE* lines was also evident. By day 7 after sowing, the germination rate of OE lines exceeded 60%, while that of WT was less than 30% (**Figure 6A**). The root length and cotyledon greening degree of germinated seedlings were also significantly better than those of the controls. Salt stress induced oxidative stress in all plants but to varying degrees. Compared with wild-type (WT) plants, OE lines showed reduced malondialdehyde (MDA) content (**Figure 6B**) and elevated soluble sugar levels (**Figure 6C**), indicating milder cell membrane damage. Consistently, proline content and peroxidase (POD) activity increased significantly in OE lines (**Figures 6D, E**), which greatly strengthened osmotic adjustment and antioxidant capacity. Moreover, chlorophyll content was restored in OE lines under salt stress (**Supplementary Figure 2a**). Moreover, under normal growth conditions, the expression differences of these genes among *OE*, *tcp10*, and WT were minimal. It further activated or reinforced a broad defensive gene expression network, mainly including: ion transport and compartmentalization genes SOS1 (**Figure 6F**); osmotic stress response protein components (**Figure 6G**); rapid proline synthesis genes P5CS1 (**Figure 6H**); and reactive oxygen species scavenging and stress protection genes APX1 (**Figure 6I**). This regulatory network suggests that *CqTCP11* may occupy an upstream hub position in salt signal transduction. These results indicate that heterologously expressed *CqTCP11* can act as a transcriptional regulator, activating multiple endogenous stress defense pathways in *Arabidopsis*, including ion compartmentalization and oxidative stress alleviation, thereby synergistically improving plant stress tolerance from seed germination to seedling growth.

### 3.8 *CqTCP11* enhances seed germination under drought stress

**Figure 7:**
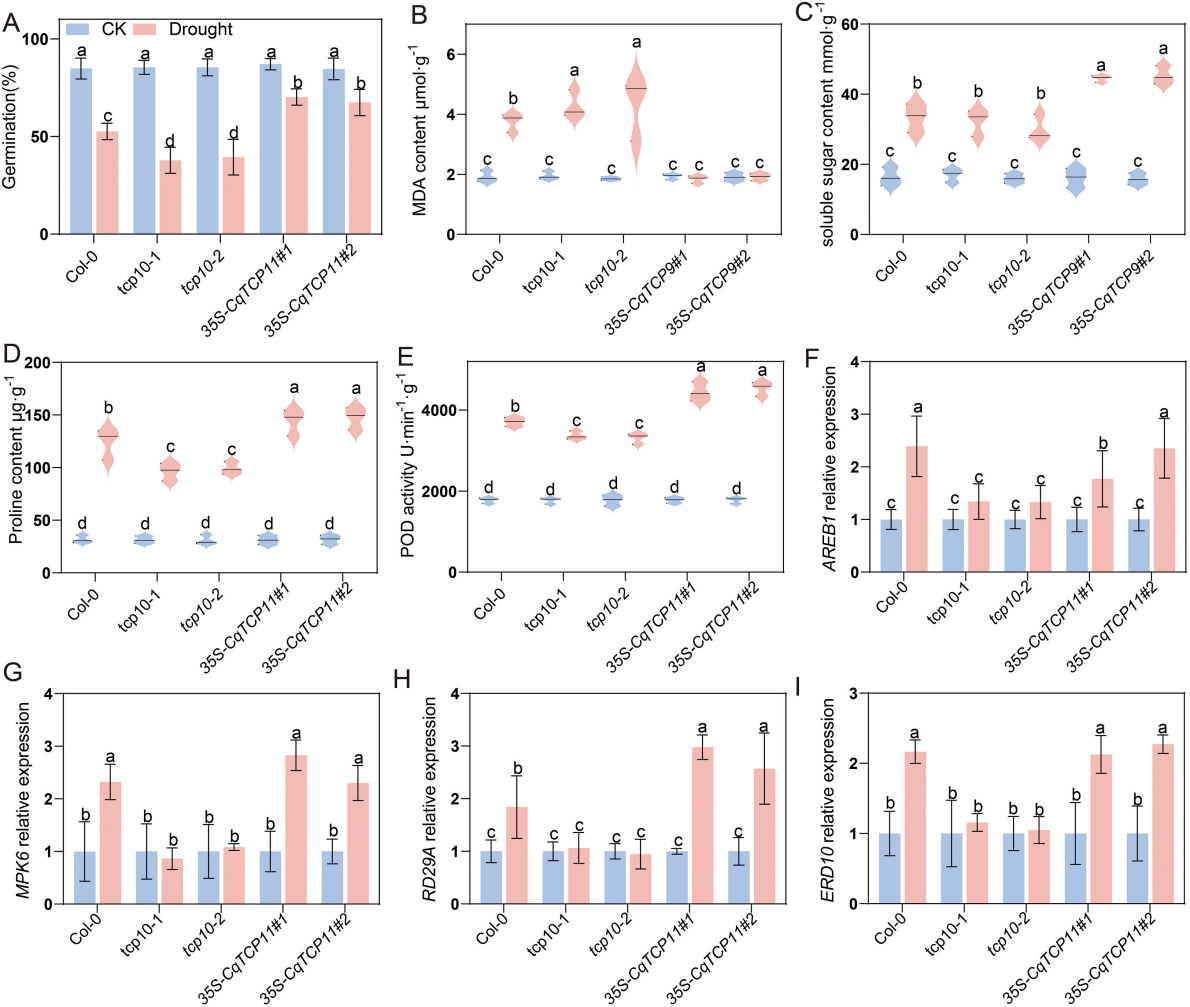
Involvement of CqTCP11 in quinoa seed drought stress tolerance. **(A)** Germination rates of *Arabidopsis* seeds of wild-type (WT), *tcp10-1*, *tcp10-2*, *35S-CqTCP11#1*, and *35S-CqTCP11#2* lines on 1/2 MS medium with or without 100 mM mannitol were counted after 7 days of culture under 16h light/8h dark (23 °C/20 °C) conditions. Data represent the mean ± SD of at least five independent replicate experiments. **(B-E)** Measurement of malondialdehyde (MDA) content **(B)**, soluble sugar **(C)**, proline (Pro) accumulation **(D)**, and peroxidase (POD) activity **(E)** in seedlings after drought stress (100 mM mannitol treatment for 3 days). **(F-I)** qRT-PCR analysis of the expression of several stress-related genes after 6 hours of drought stress. At least three independent replicate experiments were performed. Different lowercase letters indicate significant differences at *P* < 0.05 based on one-way ANOVA and Tukey’s *post-hoc* test.

Under simulated drought conditions (100 mM mannitol), seeds of *CqTCP11*-overexpressing (*OE*) lines exhibited significantly higher germination rates and faster germination speed compared to wild-type (WT). Statistical results showed that in the later stages of stress treatment, the germination rate of *OE* seeds remained consistently and significantly higher than that of WT and *tcp10* mutants, indicating that *CqTCP11* can effectively alleviate the inhibition of drought on the seed germination process (**Figure 7A**). Analysis of physiological indicators in germinated seedlings showed that under drought stress, overexpression (OE) lines exhibited markedly lower MDA content than WT plants (**Figure 7B**), with higher accumulation of soluble sugars and proline (**Figures 7C, D**). Meanwhile, OE lines maintained stronger POD antioxidant enzyme activity (**Figure 7E**), and their chlorophyll content was restored under drought stress (**Supplementary Figure 2b**). These results indicate that *CqTCP11* establishes a better physiological protective state for seedlings by enhancing osmotic adjustment capacity and reducing oxidative damage. qRT-PCR analysis revealed that under drought stress, the expression of *CqTCP11* itself was significantly upregulated in *OE* lines. Simultaneously, a series of downstream stress-responsive genes were specifically activated or enhanced, including genes potentially involved in the ABA signaling pathway AREB1 (**Figure 7F**) and MAPK signaling pathway (**Figure 7G**), as well as osmoprotectant synthesis genes (**Figures 7H, I**). This expression profile confirms that *CqTCP11* is located upstream in drought signal transduction, programming cellular stress adaptation by coordinating the gene expression of multiple tolerance pathways. As a core transcriptional regulator, *CqTCP11* multi-dimensionally enhances quinoa survival and establishment ability under drought stress by promoting seed germination vigor, strengthening the osmotic and antioxidant physiological barriers of seedlings, and activating a broad drought adaptation gene expression program.

## 4 Discussion

The TCP (TEOSINTE BRANCHED 1/CYCLOIDEA/PCF) transcription factor family, as a class of plant-specific regulatory proteins, plays key roles in plant growth, development, morphogenesis, and stress responses. In this study, we conducted a genome-wide systematic identification of the *TCP* gene family in quinoa. A total of 20 *CqTCP* genes were identified, among which 11 members were newly discovered compared with previous studies (Bai et al., 2026). Furthermore, we performed systematic bioinformatics analysis, expression pattern analysis and preliminary functional exploration to elucidate their roles in stress responses. The identification of 20 *CqTCP* genes in quinoa in this study is fewer than in monocot maize (46) and polyploid wheat (66) (Ding et al., 2019; Zhou et al., 2018) Through phylogenetic analysis (**Figure 1a**), CqTCPs were divided into three conserved subfamilies: PCF, CIN, and CYC/TB1. The PCF subfamily had the most members, consistent with the distribution trend in *Arabidopsis*, oat, and other species (Pan et al., 2024), suggesting this subfamily may have broad and fundamental functions in basic growth and development regulation. Gene structure analysis revealed that most *CqTCP* genes (approximately 75%) lacked introns or had very few introns (**Figures 2a–c**). This characteristic shares similarities with, but also differs from, oat *TCP* genes (approximately 39% intronless) and quinoa *ACS* genes (intron numbers ranging from 1 to 5) (Yin et al., 2023). Intronless or intron-poor structures are often associated with rapid transcriptional responses, which may be one of the molecular bases enabling *TCP* genes to quickly respond to environmental stimuli (e.g., low nitrogen, drought, heavy metal stress). Conserved motif analysis found that all CqTCP proteins contained motif 1 and motif 2, which constitute the core of the characteristic bHLH domain of the TCP family. Members of different subfamilies exhibited differences in C-terminal motif composition (**Figure 2c**); for example, some CIN subfamily members contained unique phosphorylation site motifs. These structural differences may relate to protein stability, subcellular localization, and interactions with other proteins, leading to functional diversification, similar to the mechanism where ACS proteins are divided into different types based on C-terminal domains and regulated by different post-translational modifications.

Analysis of cis-acting elements in the promoter regions of *CqTCP* genes indicated an enrichment of elements related to abiotic stress response, hormone signaling, and light response (**Figure 3**). Among them, abscisic acid-responsive elements (ABRE), hormone-responsive elements (DRE/CRT), low-temperature, and light-responsive elements appeared at high frequencies (Alabd et al., 2023; Nakashima and Yamaguchi-Shinozaki, 2013). This result is highly consistent with the feature of quinoa *SPL* gene promoters being rich in stress-responsive elements (Ren et al., 2022), collectively revealing the pre-transcriptional regulatory preparedness of quinoa stress-responsive genes. The presence of these elements strongly suggests that *CqTCP* genes may be widely involved in quinoa regulatory network for coping with drought, salinity, low temperature, and heavy metal stress. For example, in *Arabidopsis* cadmium stress studies, the upregulation of various stress-responsive genes *AtPCS1* and *AtABCC3* is key to plant activation of detoxification and tolerance mechanisms (Brunetti et al., 2015; Wojas et al., 2010). Similar elements in *CqTCP* gene promoters predict that they may function as central switches through transcriptional regulation in quinoa response to heavy metal stresses like cadmium. Based on transcriptome data and qRT-PCR validation, this study analyzed the expression patterns of *CqTCP* genes in multiple tissues and under different abiotic stresses (**Figures 4B, C**). *CqTCPs* exhibited significant tissue-specific expression (**Figure 4A**); for instance, some genes were highly expressed in root tips, inflorescences, or young leaves, which may be related to their functions in specific organ development, similar to the specific expression of certain *ACS* genes in flower and fruit development (De Azevedo Souza et al., 2008; Yin et al., 2023). More importantly, various stress treatments could significantly induce or repress the expression of specific *CqTCP* genes. For example, genes such as *CqTCP4*, *CqTCP5*, and *CqTCP11* showed significant upregulation in root, leaf, and seed tissues under mannitol-simulated drought and NaCl salt stress. This expression pattern is similar to the rapid induction of defense genes like *CAT1* and *P5CS1* in *Arabidopsis* plants overexpressing the *moso bamboo TCP11* gene under salt stress (Xu et al., 2022). These stress-responsive *CqTCP* genes may act as transcriptional regulators, activating or repressing the expression of a series of downstream stress-responsive genes, thereby coordinating plant adaptive responses. For instance, they may regulate the expression of antioxidant enzyme system genes, osmolyte (e.g., proline, soluble sugar) synthesis-related genes, or ion transporter (e.g., ABC transporter) genes, which are core physiological and biochemical mechanisms for plants to cope with heavy metal and other stresses (Liu et al., 2024; Song et al., 2024). Through heterologous overexpression in *Arabidopsis*, we confirmed that *CqTCP11* can confer stronger tolerance not only during the vegetative growth stage but also, importantly, during seed germination—the most vulnerable and environmentally constrained key stage of the plant life cycle. Its ability to significantly improve seed germination rate and early radicle growth under both drought and salt stress strongly suggests that this gene plays an upstream “master switch” regulatory role in quinoa adaptation strategy to complex environmental pressures (**Figure 6A**). The significantly reduced MDA content in *OE* lines under salt and drought stress is direct evidence of less damage to their membrane systems(**Figures 7A-E**). This benefits from the preemptive or enhanced activation of the antioxidant enzyme system (POD, SOD), maintaining cell membrane integrity and reducing oxidative damage (Panfili et al., 2019; Wang et al., 2020). Reactive oxygen species (ROS) burst is a key factor in stress-induced inhibition of germination, attacking membrane lipids, proteins, and nucleic acids (Li et al., 2023). *CqTCP11* may construct a more efficient ROS scavenging system by upregulating antioxidant enzyme gene expression, thereby protecting the integrity of key organelles (e.g., mitochondria, plasma membrane) required for germination, providing a fundamental guarantee for radicle protrusion and cell elongation (J. Chen et al., 2023; Y. Wang et al., 2025). The significant accumulation of proline and soluble sugars is an important strategy for *OE* lines to cope with drought and salt stress. Proline is not only an efficient osmolyte but also acts as a radical scavenger and protein stabilizer (Furlan et al., 2020; Ren et al., 2018). Soluble sugars directly provide carbon skeletons and energy for respiration and biosynthesis during seed germination. These enhance osmotic adjustment and energy homeostasis. *CqTCP11* may promote the expression of genes related to sugar metabolism and proline synthesis, thereby maintaining sufficient turgor pressure and metabolic activity in embryonic cells when external water potential decreases or under ionic stress. This is the driving force source for the radicle to overcome external mechanical resistance and ion toxicity for elongation.

Although seed germination initially relies on stored reserves, the rapid establishment of photosynthetic capacity after cotyledon expansion is crucial for seedling survival. The ability of *OE* lines to maintain higher chlorophyll content under stress indicates that *CqTCP11* expression helps protect or delay chloroplast degradation. This may be achieved indirectly through the antioxidant system or possibly via direct regulation of chlorophyll metabolism-related genes, ensuring that post-germination seedlings can quickly transition to photoautotrophy, thereby successfully completing the shift from heterotrophy to autotrophy and improving establishment success (Zhou et al., 2018). The results of this study form an interesting parallel with research on the quinoa *CDK* gene family. The promoters of genes from both families are enriched with stress-responsive elements such as ABRE and DRE (Wang et al., 2023). We speculate that *CqTCP11* itself may be induced by signals like ABA and drought, and subsequently, its encoded protein, acting as a transcription factor, further binds to TCP binding elements (e.g., GGNCCCAC) in the promoters of downstream target genes or interacts with other stress-responsive elements, thereby extensively reprogramming the stress-responsive gene network. However, further experiments such as ChIP assays are required for solid validation. Its regulated network may broadly cover multiple functional modules mentioned earlier, including antioxidation, osmotic adjustment, ion transport, and photoprotection. This “one transcription factor regulates a cluster of functional modules” mode enables efficient coordination of multifaceted physiological and biochemical responses, rapidly establishing systemic stress resistance during the critical, time-limited, and resource-constrained window of seed germination.

## 5 Conclusion and prospects

In summary, this study presents the genome-wide identification and bioinformatics analysis of the *TCP* gene family in quinoa, revealing its evolutionary characteristics, structural diversity, and great potential for responding to abiotic stress. Several candidate genes including *CqTCP11* and *CqTCP18* were preliminarily screened via expression analysis in response to drought and salt stress. The functions of these genes are analogous to the role of quinoa ACS family members in ethylene-mediated stress responses, but as transcription factors, they occupy a higher regulatory hierarchy, and their action networks are likely more complex. Future research should focus on in-depth functional validation of these key candidate *CqTCP* genes. This can be achieved by constructing genetic materials to systematically analyze their physiological phenotypes and biochemical indicator changes under specific stresses (especially salinity, drought, and potential heavy metal stress common for quinoa) and to elucidate their regulated downstream gene networks. This will not only help reveal the unique functions of TCP transcription factors in quinoa, this star stress-tolerant crop, providing important gene resources for improving quinoa environmental adaptability through molecular breeding, but also enrich our understanding of the universal mechanisms of the plant TCP family in abiotic stress responses. This study localized the function of quinoa-derived *CqTCP11* in the model plant to the specific and critical developmental stage of seed germination, associating it with systematic physiological indicator changes, providing a new dimension for understanding TCP family functions in plant stress adaptation. The results confirm the conserved and effective function of this gene across species, offering a promising candidate gene for stress resistance genetic improvement of crops, particularly quinoa itself. Its potential to improve seedling emergence rates in polluted or dry soils holds practical application value.

## Data Availability

The datasets presented in this study can be found in online repositories. The names of the repository/repositories and accession number(s) can be found in the article/**Supplementary Material**.
