# Supplementary material for: Expression of the quinoa TCP gene family and analysis of CqTCP11 involvement in seed germination stress response
Source: Front Plant Sci. 2026 Jun 2;17:1860606. doi: 10.3389/fpls.2026.1860606 (PMC13269216; doi:10.3389/fpls.2026.1860606)

**Figure S1 Chromosome distribution of *TCP* gene family members in Chenopodium quinoa**

A total of 20 quinoa TCP genes (*CqTCP1*–*CqTCP20*) show their physical distribution on different chromosomes of the quinoa genome. The scale bar on the left indicates the physical length of chromosomes (unit: Mb). The yellow texts represent the scaffold numbers of the quinoa genome, and the black vertical lines stand for scaffold sequences. The red texts are the names of each *CqTCP* gene, and the labeled positions correspond to the physical loci of genes on the chromosomes.


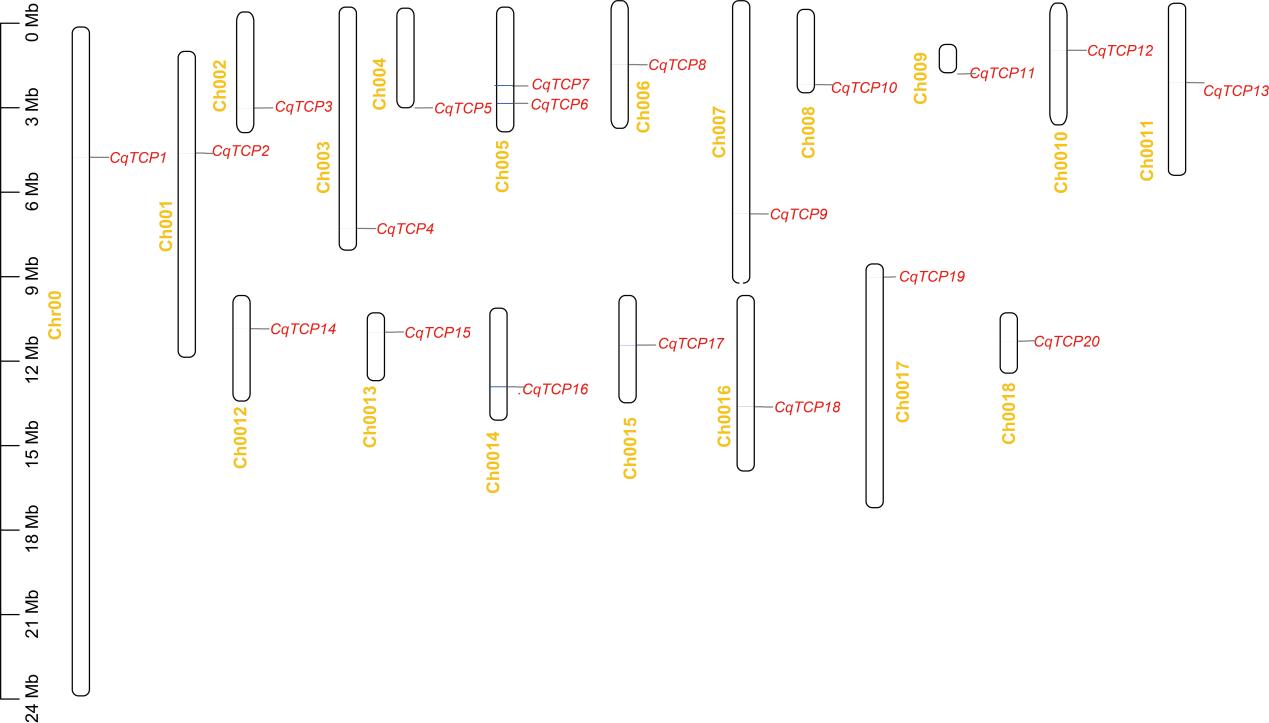


**Figure S2 Chlorophyll content under salt and drought stress**

Chlorophyll content was measured in wild-type (WT), *tcp10-1*, *tcp10-2*, *35S-TCP11#1*, and *35S-TCP11#2* *Arabidopsis* plants cultured for 7 days under 16 h light/8 h dark (23°C/20°C) conditions in 1/2MS medium with or without salt (a) and under drought stress (b). Salt stress was induced with 100 mM NaCl, and drought stress with 100 mM mannitol. Experiments were performed in at least three independent replicates. Lowercase letters denote groups with statistically significant differences (*p* < 0.05, Student's t-test).


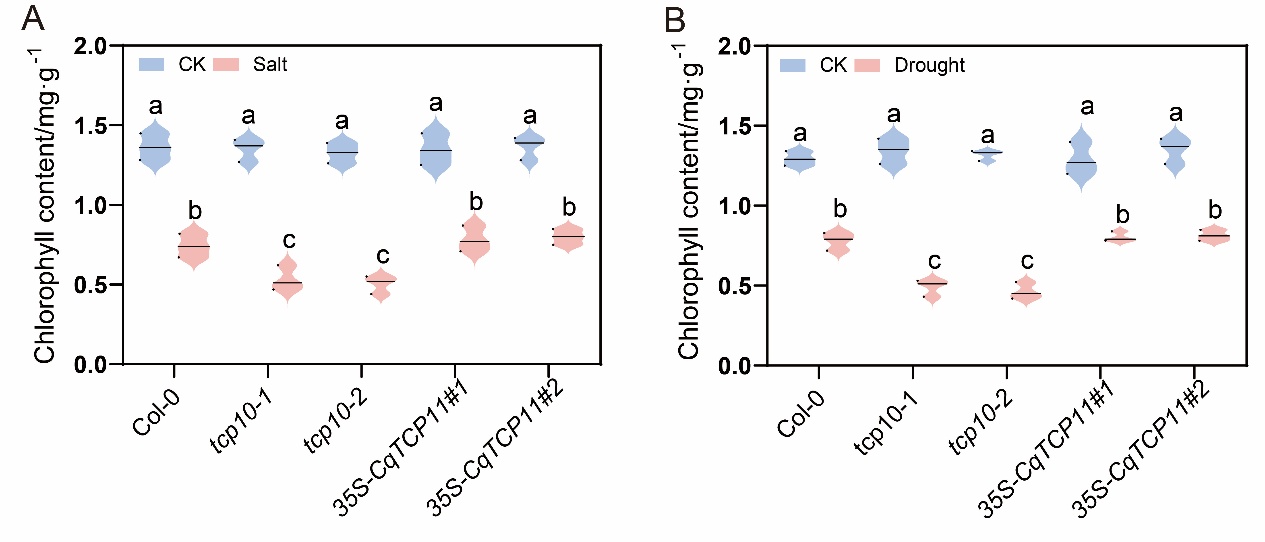

Supplement: Supplementary file 1 [file SupplementaryFile1.docx]
